# Editorial: Neurological Disorders and COVID-19: Interconnections, Molecular Links, and Therapeutic Perspectives

**DOI:** 10.3389/fmed.2022.928912

**Published:** 2022-05-31

**Authors:** Suliman Khan, Ghulam Nabi, Muhammad Wajid Ullah

**Affiliations:** ^1^Department of Cerebrovascular Diseases, The Second Affiliated Hospital of Zhengzhou University, Zhengzhou, China; ^2^Department of Medical Laboratory Technology, The University of Haripur, Haripur, Pakistan; ^3^Institute of Nature Conservation, Polish Academy of Sciences, Krakow, Poland; ^4^Biofuels Institute, School of the Environment and Safety Engineering, Jiangsu University, Zhenjiang, China

**Keywords:** SARS-CoV-2, brain and nervous system diseases, treatment and prevention, molecular mechanisms, therapeutics

In addition to respiratory symptoms, severe acute respiratory syndrome (SARS-CoV-2) also causes neurological complications (1). SARS-CoV-2 might enter the central nervous system (CNS) through the neural–mucosal interface (olfactory tract of CNS), as indicated by the change in smell and taste perception in COVID-19 patients. The transportation of SARS-CoV-2 into the CNS might be facilitated by leukocytes and CNS endothelia. Nevertheless, it has been widely accepted that COVID-19 increases the risk of neurological complications such as myalgia, dizziness, headache, ageusia, stroke/intracerebral hemorrhages, etc., as reported in most hospitalized COVID-19 patients (2).

It is yet to unveil how SARS-CoV-2 affects the neurological system; however, the pathophysiology of SARS-CoV-2 offers clues to understanding the possible mechanisms of neurological damage and associated complications. The viral entry into the cell through ACE2 receptors can trigger the pro-inflammatory and pro-coagulable reactions through vascular integrity disruption and clotting cascade activation. This phenomenon can further disrupt the blood pressure autoregulation, that in turn increases the risk of stroke and associated neurological complications (1). Furthermore, neurovirulence may occur in response to the SARS-CoV-2-mediated prothrombotic effect on inflammatory responses, immunological responses, and CNS vasculature. Besides, the olfactory sensory neurons are thought to play an important role in entrance and virulence in the brain stem, basal ganglia, and thalamus. The development of hyposmia confirms the involvement of olfactory epithelium in viral entry into the brain (3). The disruption of the blood-brain-barrier (BBB) is likely to serve as the potential route for viral entry into the brain, where it travels through cerebral circulation and damages the capillary epithelium. Here, the expression of ACE-2 by cells of BBB facilitates the virus transport to CNS and increases the risk of edema and endothelial damage (4).

Severe COVID-19 disease mediates pro-inflammatory responses by producing interferon (IFN)-α, tumor necrosis factor (TNF)-γ, monocyte chemotactic protein (MCP)-1/C-C motif, chemokine ligand (CCL)-2, vascular endothelial growth factor (VEGF), granulocyte colony-stimulating factor, and different interleukins (ILs), which lead to acute respiratory distress syndrome. However, in some cases, COVID-19 causes multiorgan dysfunction without the involvement of cytokines (5). IL-1R induces the secretion of IFN-γ and IL-6, which leads to T cell recruitment and macrophage activation at the site of infection. Collectively, these cytokines induce inflammation and cause the destruction of lung parenchyma (6).

Considering the neurological impact of COVID-19, this Research Topic in Frontiers in Medicine, Sections “Infectious Diseases—Surveillance, Prevention and Treatment” entitled “*Neurological Disorders and COVID-19: Interconnections, Molecular Links, and Therapeutic Perspectives*” is aimed to study the neurological consequences of COVID-19 and effective therapeutic options. The articles covered by this Research Topic mainly focus on establishing an association between COVID-19 and neurological disorders by studying the role of different molecules, pathogenicity, ischemic effect, and the impact of social media on mental health during the pandemic of COVID-19.

Little is known about the neurological manifestation of SARS-CoV-2: whether it directly attacks the CNS or activates the peripheral nervous system and immune cell infiltration. To this end, Almutairi et al. reviewed the molecular aspects of cellular mechanisms associated with COVID-19-induced neuroinflammation. During COVID-19 infection, the immune system is activated by high concentrations of chemokines, cytokines, and free radicals at BBB. Disruption of BBB leads to infiltration of inflammatory and immune system-associated cells (such as T-cells) into the CNS and activates the immune resident cells, such as microglia and astrocytes. A perspective study by Mehboob et al. elaborated the inflammation mechanism during infection of COVID-19 and its association with trigeminal ganglion (TG). After entering the cells, the virus resides in TG and utilizes its peptides, such as the Substance P (SP). The release of SP under noxious stimulus activates the blood vessels and thus initiates a cytokine storm that causes respiratory distress. In normal circumstances, SP regulates several pathological and physiological functions; however, its production in high concentration disturbs many biological functions and thus should be blocked by the neurokinin-1 receptor (NK-1R) antagonist. This could be a perspective approach to treating COVID-19 and should be verified through experimentation. Besides directly affecting the individuals by causing respiratory disorders, COVID-19 may also worsen the symptoms of other diseases and lead to severe illness. For instance, a study by Wang et al. investigated the potential development of acute ischemic stroke (AIS) in ischemic stroke patients infected with COVID-19. The patients with prior stroke showed a high proportion of developing AIS by demonstrating critical illness and mortality with a high death risk in premorbid modified Ranking Scale (MRS) and old people. This study provides a basis for understanding the association between ischemic stroke and COVID-19, which might apply to other illnesses, thus necessitating the management of patients with underlying medical issues to minimize the death risk.

In addition to impacting the environment (7), maternity and child care (8), education (9), business and economy (10), and other walks of life, the pandemic of COVID-19 has severely affected mental health. The knowledge, attitude, and perceptions (KAPs) play a vital role in developing and implementing standard operating procedures (SOPs) for containing the contagion and minimizing the damage. A couple of studies in this Research Topic assessed the KAPs in selected populations toward COVID-19. In one study, Khattak et al. carried out an online cross-sectional study and assessed the KAPs in a Pakistani population (aged ≥18 years) toward COVID-19. Statistical analysis of collected data showed good awareness, reasonable attitude, and perceptions toward COVID-19. A major limitation in this study is that the respondents were only the social media users who have better access to the knowledge about COVID-19 and the general population may behave differently. In a related study, Rizwan et al. studied the impact of social media use on psychological distress and KAPs of COVID-19 in selected social media users, both male and female, in Pakistani population. The results showed that compared to males, the female social media users were more vulnerable to various psychological distresses. The male participants were more aware of COVID-19, while the female participants showed relatively better attitude toward COVID-19 and practiced precautionary measures. These two studies necessitate the launching of awareness programs in general public to fill the gap between KAP toward COVID-19.

This Research Topic not only overviews the clinical aspects and association of COVID-19 with neurological disorders but also discusses the impact of social media on the mental health of individuals during the pandemic of COVID-19, which on the one hand, provides awareness to people about the pandemic situation but on the other hand is a major reason for the development of psychological distress.

## Author Contributions

SK and MWU drafted and proofread the manuscript. SK, GN, and MWU edited and modified the manuscript. All authors contributed to the article and approved the submitted version.

## Conflict of Interest

The authors declare that the research was conducted in the absence of any commercial or financial relationships that could be construed as a potential conflict of interest.

## Publisher's Note

All claims expressed in this article are solely those of the authors and do not necessarily represent those of their affiliated organizations, or those of the publisher, the editors and the reviewers. Any product that may be evaluated in this article, or claim that may be made by its manufacturer, is not guaranteed or endorsed by the publisher.
